# Emotional Intelligence Scale for Male Nursing Students and Its Latent Regression on Gender and Background Variables

**DOI:** 10.3390/healthcare10050814

**Published:** 2022-04-27

**Authors:** Jiunnhorng Lou, Hsiaochi Chen, Renhau Li

**Affiliations:** 1 Department of Nursing, Hsin Sheng College of Medical Care and Management, Taoyuan 325004, Taiwan; stujhl@gmail.com (J.L.); a9104059@gmail.com (H.C.); 2 Clinical Psychological Room, Chung Shan Medical University Hospital, Taichung 40201, Taiwan; 3 Department of Psychology, Chung Shan Medical University, Taichung 40201, Taiwan

**Keywords:** male nursing students, emotional intelligence, latent regression, gender, scale

## Abstract

This study aimed to develop an emotional intelligence (EI) scale for male nursing students and investigate its associations with gender, age, religious beliefs, and father’s and mother’s education level. We recruited 384 male nursing students in Taiwan to construct an EI scale comprising 16 items with four factors: recognizing the emotions of others, emotional self-awareness, self-emotional expression, and self-emotional management. The scale had factor loadings of 0.64−0.80. The reliability coefficients for the subscales ranged from 0.80 to 0.84, while that for the total scale was 0.93. We also recruited 402 female nursing students for comparison. Latent multiple regression of the EI factors showed that male students had higher self-emotional expression but lower self-emotional management than females. Age was negatively associated with self-emotional management for both genders. Religious beliefs were negatively associated with emotional self-awareness in male students, and with recognizing the emotions of others in females. Father’s and mother’s education had no association with EI in male students; however, father’s education was positively associated with all EI factors in females, and mother’s education was negatively associated with recognizing the emotions of others and self-emotional expression. These results provide insight into male nursing students’ EI and the background variables influencing EI.

## 1. Introduction

The World Health Organization [1] reported that only 10% of nursing professionals worldwide are male, with some variation in rates across regions and countries. Although the number of men entering the nursing profession is increasing, societal stereotypes and the lack of male role models in nursing may have a negative impact on male nursing students’ motivation, thereby affecting their emotional intelligence (EI) [2,3]. Nursing care is conditioned by gender roles and stereotypes that present men as less capable than women of experiencing and managing emotions [4]. The lack of gender diversity in the nursing profession has long been recognized as a cause for concern. Male nursing students have also reported perceived differences in clinical practice, such as feeling alienated in the obstetrics and gynecology practicum [5,6]. Therefore, promoting academic support for male nursing students may help increase the number of men registering as nurses in the workforce [7]. Moreover, nursing is a profession that requires dealing with people directly on a daily basis [8]. Therefore, EI is an important factor in both personal and professional aspects of nurses’ lives [9]. International studies have also identified that EI influences nurse–patient relationships [10].

The term “male nurse” is often problematic to those it refers to, as the label carries stereotypes that further marginalize this minority group in the nursing profession [11]. Such stereotyping not only limits the entry of men into the profession, but also leads to challenges for male nursing students in practice [12]. Studies have shown that male nursing students face more setbacks, conflict of gender roles, and negative evaluations from the public, which makes them feel lonely and alienated, thereby impacting their EI [13,14]. EI is an important factor for maintaining high-quality nurse–patient relationships [15]. Male nursing students were found to have the same EI potential as female students when they enrolled in nursing schools [2,16]. Furthermore, a study in Australia found no differences in EI between male and female nursing students [17]. However, other studies reported that female nursing students had higher EI than males [18,19]. Therefore, assessment of male nursing students’ EI needs greater consideration in nursing education [20] to observe male students’ EI levels relative to female students and to examine the association of EI with background variables.

EI is defined as the ability to recognize, produce, and manage emotions. This ability can improve awareness of self-emotion and perception of others’ emotions, thereby maintaining the appropriate use and management of emotion [21,22,23,24]. These studies identified the following sets of emotional skills to define EI: (1) the ability to accurately identify emotions in oneself and others; (2) the ability to use emotions to facilitate reasoning; (3) the ability to understand emotions in oneself and others; and (4) the ability to manage emotions in oneself and others. Moreover, EI is an important factor based on which male nursing students can develop healing relationships in the clinical context; it can also help them manage self-emotion and build excellent nurse–patient relationships [15,25,26], as well as promote positive professional performances [27].

It is usually necessary for nurses to help patients regulate negative emotions. EI is likely to influence academic and clinical practice performance across a variety of fields in health; nursing students learn and staff nurses work in a stressful environment arising from various factors such as work overload, long working hours, and having to interact with different personnel ranging from patients to healthcare teams [28,29]. Two major stressors for male nursing students—i.e., the fact of nursing being viewed as a highly feminine profession and being a minority in the nursing workforce—had negative influences on the EI of male nursing students, including the promotion of intense anger and dumpiness [30,31,32]. Some studies also indicated that the EI of male nursing students directly influenced their clinical performance, retention rate, and development of nursing careers [18,33,34,35].

Male nurses play a valuable role in healthcare, as they bring diversity, valuable skills, and unique perspectives to the nursing workforce. Therefore, as male nursing students study and train in nursing schools and become male nurses after graduating, a higher level of EI can decrease the stress of gender roles, increase long-term occupational health, enhance the ability to cope with clinical stress, and improve the quality of healthcare [36,37,38,39]. 

An increasing number of male students are enrolling in nursing schools in Taiwan [40]; however, thus far, no suitable scale has been developed to assess male students’ EI, even though scales assessing EI mainly among female nursing students have been developed [41]. The aim of the present research was to develop an EI scale for male nursing students using advanced techniques and confirmatory factor analysis, with good reliability and validity. The study also aimed to offer novel findings regarding the association of EI with gender and other background variables using latent regression analyses with the Rasch model of the item response theory. The results can help provide a better understanding of male nursing students’ EI compared with that of females, as well as of the association of EI with demographic factors.

## 2. Materials and Methods

This study involved a cross-sectional survey conducted in Taiwan. The questionnaire included the following items: gender, age, having religious beliefs or not, father’s education level, mother’s education level, general learning experiences, and a draft of the EI scale. The study was approved by the Institutional Review Board (IRB No. 202110-E101).

### 2.1. Procedures

The draft of a questionnaire comprising 26 items was first prepared by the authors based on the definitions of EI for four dimensions: “recognizing the emotions of others”, “emotional self-awareness”, “self-emotional expression”, and “self-emotional management”. After the draft was completed, five subject matter experts were invited to scrutinize the items. The purpose of the expert review was to test the content and construct validities of the scale and determine if the content adequacy, conceptual clarity, and meaning of the questions were consistent with each dimension [42]. Thereafter, the scale was formally administered to male nursing students. Data were first analyzed by a structural equation model (SEM). Next, to determine whether EI is influenced by gender, we collected data by conducting a survey on female nursing students. Finally, to examine the background factors that influence or are associated with EI, we performed a latent multiple regression of the EI total scale and its subscales on gender, age, religious beliefs, and father’s and mother’s education level. 

### 2.2. Participants

We adopted the purposive sampling method to recruit male nursing students. First, out of a total of 18 nursing schools, two nursing schools from each of the three parts of Taiwan (the north, middle, and south zones) were selected. Second, male nursing students from the six shortlisted schools were briefed about the research and were encouraged to participate. The rate of participation was over 95%. Out of these, 384 male nursing students (about 35% of the total population) who had completed the fundamental nursing practicum were recruited [43]. The participants signed the informed consent form and proceeded to respond to the questionnaire anonymously. This group was the main sample for the development of the EI scale. Their ages ranged from 18.1 to 23.5 years, with a mean of 21.00 and a standard deviation of 0.89. We also recruited a sample of 402 female nursing students from a nursing school for the purpose of comparison. They were aged from 20.0 to 24.0 years, with a mean of 20.61 and a standard deviation of 1.36. Other descriptive statistics are presented in Table 1.

### 2.3. Instrument 

We referred to EI theories and relevant empirical research on nursing students (e.g., [10,21,23,44]) to develop the EI scale with four dimensions, focusing on male nursing students. It included “recognizing the emotions of others”, “emotional self-awareness”, “self-emotional expression”, and “self-emotional management”. Recognizing the emotions of others indicates that male nursing students can understand others’ emotional states. Emotional self-awareness means that male nursing students are aware of and able to understand their own emotions. Self-emotional expression means that, when in a particular mood, male nursing students can properly perform physiological, psychological, and manifest behavior to express themselves. Self-emotional management means that male nursing students are aware of their own emotions and can manage them.

The draft of the scale contained 26 items, and the four dimensions (subscales) had 8, 6, 7, and 5 items, respectively. A five-point Likert-type scoring was used, where 1 point represented “none”, 2 represented “seldom”, 3 represented “sometimes”, 4 represented “usually”, and 5 represented “always”. Higher scores indicated higher levels of “recognizing the emotion of others”, “emotional self-awareness”, “self-emotional expression”, and “self-emotional management”; therefore, the higher the total score, the higher the level of EI. The content validity index was calculated as 0.93, based on the five experts’ scrutiny.

### 2.4. Statistical Analysis

Of the 384 male nursing students, 230 (60%) were randomly selected to test the four-factor model of the EI scale with confirmatory factor analysis using SEM. We referred to Li’s [45] item selection strategies, mainly based on a modification index involving factor loadings or item error correlations. After the final items were selected, the total male sample was used to confirm the four-factor model. LISREL 8.8 software was used; the common standards to reflect model fit are listed below (e.g., [40,46,47]): χ^2^/*df* (the ratio of chi-square to the degrees of freedom) < 5, standardized root-mean-square residual (SRMR) < 0.06, root-mean-square error of approximation (RMSEA) < 0.08, comparative fit index (CFI) > 0.90, non-normed fit index (NNFI) > 0.90, and adjusted goodness of fit index (AGFI) > 0.90.

Next, we performed a latent multiple regression analysis of the EI total scale and its four subfactors to determine the predictive effect of several relevant background variables, such as gender, age, religious beliefs, and father’s and mother’s education levels. The latent multiple regression analysis was based on the item response theory, conducted with Conquest 2.0 software.

## 3. Results

As shown in Table 1, of the 786 nursing students, 290 (36.9%) had no religious beliefs, while 496 (63.1%) had at least one religious belief. Regarding father’s and mother’s education levels, senior high school/vocational school had the highest ratios (37.7% and 37.4%, respectively), while graduate school and above (7.3% and 6.7%, respectively) and elementary school (7.4% and 6.7%, respectively) had the lowest.

The four-factor model with 26 items was first tested using confirmatory factor analysis. The model fit indices showed χ^2^ = 695.35, *df* = 293, *p* < 0.001, χ^2^/*df* = 2.37, CFI = 0.98, NNFI = 0.98, AGFI = 0.85, SRMR = 0.043, and RMSEA = 0.060, indicating poor model fit. The factor loadings ranged from 0.63 to 0.79, with a mean factor loading of 0.70. The correlation coefficients among the four factors ranged from 0.81 to 0.87. The Cronbach’s alpha reliability coefficients were 0.95 for the total scale and 0.82−0.90 for the four subscales. To promote the convenient use of the scale and increase the model fit to avoid validity shrinkage in applied research, we judged that fewer and better items would be suitable. Therefore, six items were deleted for high correlation between measurement errors, three items were deleted for loading on non-principal factors, and one item was deleted for low factor loading. Finally, 16 items were selected based on the modification index of the SEM. The model fit indices for the four-factor model with 16 items were χ^2^ = 207.60, *df* = 98, *p* < 0.001, χ^2^/*df* = 2.12, CFI = 0.99, NNFI = 0.98, AGFI = 0.91, SRMR = 0.037, and RMSEA = 0.054, indicating better model fit outcomes. 

Table 2 shows the item contents and the factor loadings, ranging from 0.64 to 0.80, with a mean factor loading of 0.72, showing better convergent validity. The Cronbach’s alpha coefficients were 0.93 for the total scale and 0.80−0.84 for the four subscales, revealing high internal consistency reliability. The correlation coefficients among the four factors ranged from 0.76 to 0.84, showing discriminant validity by a chi-square difference test between nested models. Furthermore, a second-order factor analysis also showed a good model fit result (χ^2^ = 211.25, *df* = 100, *p* < 0.001, χ^2^/*df* = 2.11, CFI = 0.99, NNFI = 0.98, AGFI = 0.91, SRMR = 0.038, and RMSEA = 0.054), indicating an EI factor underlying the four factors. In addition, the correlation coefficients among the four subscales of the EI scale showed plausible correlation sizes (0.63−0.70) for a meaningful sum of the EI scale items to use.

Table 3 shows the results of the latent multiple regression of the EI total scale factor and its four subfactors on gender, age, religious beliefs, and father’s and mother’s education level. Male students had higher self-emotional expression (b = 0.189, *p* < 0.05) but lower self-emotional management (b = −0.227, *p* < 0.01) than females. Older nursing students had lower self-emotional management than younger students (b = −0.124, *p* < 0.001). Students with religious beliefs scored lower than those without religious beliefs on the total scale and the four subfactors, except for self-emotional management (b = −0.156 for the total scale; b = −0.230, −0.158, and −0.196, respectively, for the subscales; *ps* < 0.05). The nursing students whose fathers had higher education levels showed significantly higher total scale and subscale scores (b = 0.135; 0.204, 0.142, 0.152, and 0.107, respectively, *ps* < 0.05) than those whose fathers had lower education levels. However, mothers’ educational levels showed no significantly predictive effect on the total scale and all four factors. 

Table 4 shows the latent multiple regression of the EI total scale factor and its four factors on age, religious beliefs, and father’s and mother’s education level separately for the male and female nursing students. Age was negatively associated only with self-emotional management for both genders (b = −0.153, *p* < 0.05; b = −0.111, *p* < 0.001). Religious beliefs had a negative association with emotional self-awareness for male students (b = −0.329, *p* < 0.05) and with recognizing the emotions of others for females (b = −0.194, *p* < 0.05). Furthermore, for male students, father’s and mother’s education level had no significant association with any of the EI factors, whereas, for females, father’s education level had a positive association with all of the EI scales (b = 0.212 for the total scale; b = 0.163, 0.238, 0.132, and 0.169, respectively, for the subscales; *p* < 0.01); moreover, mother’s education level had a negative association with recognizing the emotions of others (b = −0.127, *p* < 0.01) and with self-emotional expression (b = −0.105, *p* < 0.05).

## 4. Discussion and Suggestions

### 4.1. Discussion

As for the latent multiple regression of the EI total scale factor and its four subfactors on gender and other background variables, it was executed under the item response theory with two analyses: one for the EI total scale factor, and the other for its four subfactors. The EI total scale factor was defined as a single factor underlying the 16 items of the EI scale. This is consistent with the assumption of unidimensionality in the item response theory. By a confirmatory factor analysis with a first-order one-factor model using the total sample size of 786, we obtained a good model fit (χ^2^ = 505.09, *df* = 104, *p* < 0.001, χ^2^/*df* = 4.86, CFI = 0.97, NNFI = 0.97, AGFI = 0.90, SRMR = 0.043, and RMSEA = 0.070), which confirmed the assumption. Furthermore, as a development of the item response theory, simultaneous conduction with multiple dimensions was also available [48]. Using the total sample size, by a confirmatory factor analysis with a first-order four-factor model, we again obtained a good model fit (χ^2^ = 281.70, *df* = 98, *p* < 0.001, χ^2^/*df* = 2.87, CFI = 0.99, NNFI = 0.98, AGFI = 0.94, SRMR = 0.034, and RMSEA = 0.049). Therefore, it was meaningful to perform the latent multiple regression analyses. It is noteworthy that the variables were analyzed as latent variables, and not as observed variables as in traditional regression analyses. The advantage of this method is that data can be transformed from ordinal Likert-type scales into continuous scales, and regression analyses can be performed simultaneously without the interference of measurement errors [49], making the analysis more precise and reliable. 

In the present research, no gender differences in the EI total scale factor were found, which is consistent with existing research [17]. However, additional investigations were conducted for the four subfactors, and it was revealed that although there were no gender differences in recognizing the emotions of others or in the aspect of emotional self-awareness, male nursing students had significantly higher self-emotional expression and lower self-emotional management compared to female nursing students. These results indicate that male nursing students had possibly to face more obstacles at work [13,14,50] and, therefore, had considerably more opportunities to express their emotions (higher self-emotional expression). However, this emotional self-expression might seem to be inadequate or inappropriate at times (lower self-emotional management), as reported in past research [31,32].

In addition, the present study revealed a novel finding, undetected in past research: for both genders, age was significantly negatively associated with self-emotional management, despite showing no association with the EI total scale factor. This may imply that, as nursing students age, they face higher levels of stress from preparing to enter their nursing career, resulting in lower control and management of emotions. Specifically, the participants in this study had all completed the practicum of fundamental nursing and were aware of the challenges they would face in their future nursing careers, which may have caused stress. 

In addition, religion is usually viewed as a cultural system that may actively shape the emotions of adherents, and the religion–emotion relationship may be modified by cultural or regional contexts [51]. In Taiwan, religious beliefs are closely combined with the traditional Chinese culture, which emphasizes calmness, collectivism, and conservatism. Therefore, nursing students with religious beliefs may have scored lower on recognizing the emotions of others because of emphasizing conservatism, revealed lower emotional self-awareness because of emphasizing collectivism, and had lower self-emotional expression because of emphasizing calmness, resulting in a lower EI total scale score. However, further investigation is needed to explain why male nursing students with religious beliefs had significantly lower emotional self-awareness than those without religious beliefs, and why female students with religious beliefs scored significantly lower on recognizing the emotions of others than those without religious beliefs. Nonetheless, it can be speculated that male nursing students with religious beliefs had a tendency towards collectivism, such as spending more time together to face the challenges in the nursing field, and that female students with religious beliefs had a tendency towards conservatism, such as fulfilling one’s nursing duties faithfully and uncomplainingly.

Finally, the results showed that, in general, father’s education level, rather than mother’s, was significantly positively associated with the EI total scale factor and its four subfactors. However, for male nursing students, none of the EI factors was associated with either father’s or mother’s education level; for the female students, all EI factors were positively associated with the father’s education level, whereas, recognizing the emotions of others and self-emotional expression were negatively associated with the mother’s education level. This indicates that the female nursing students’ EI was influenced positively by their fathers’ education level and negatively by their mothers’ education level to a greater extent compared with the male students. It is possible that, as men, male nursing students are nurtured more openly and rendered more rights to future choices regardless of their father’s or mother’s education levels; thus, as shown in this study, they have higher self-emotional expression but lower self-emotional management than females. However, the present study design did not permit experimental manipulation; further research is required to confirm the results.

### 4.2. Limitations and Suggestions

The present study has limited generalizability due to the sampling method and sample size used. For the most part, it was a quantitative study with limited qualitative item-making processes. The main study sample comprised 384 male nursing students and as such does not seem to be sufficiently large for developing a scale, much less to analyze it using advanced statistical methods. However, it may be noteworthy to point out that the average number of male nursing students enrolled every year in Taiwan is approximately 550. Out of this, the population that had finished the fundamental nursing practicum was approximately 1100—about 35% of the total population that may be considered adequately representative [43]. Moreover, the distribution of religious beliefs and parents’ education levels were consistent between male and female students as per the chi-square test. Therefore, the sample of female nursing students was also representative of the population, despite being recruited from a single nursing school.

Additionally, for item-making and data collection, future researchers may conduct qualitative or mixed-method research using a new and comprehensive phenomenological method called Online Photovoice (OPV) [52,53] to understand what EI means for male nursing students and others. The themes emerging from such a study may reveal new important aspects of EI that the scales may have missed. OPV gives an opportunity to participants to state their own experience with as little manipulation as possible, if at all, as compared to traditional quantitative methods. In addition, further researchers may also consider Community-Based Participatory Research (CBPR) as one way to develop more items for the EI scale [54]. CBPR may also be used to collect data from diverse populations and to adapt and validate scales for different populations.

Notably, although the present study did not perform experimental manipulation to determine cause–effect relationships, its findings are based on advanced statistical methods involving latent variables and, therefore, may be considered more valid and reliable than previous findings. The results can be valuable for future research and guidance for male nursing students. As a result, future researchers may use the EI scale to execute several valuable studies, such as comparing the EI of nursing students with that of working nursery staff who have lesser or more experience; exploring gender-related features of the connection between EI and performance of nursing students during online learning [55]; or using EI as an indicator of nursing students’ readiness for professional growth and the like [56].

## 5. Conclusions

In this study, we developed an EI scale for assessing the EI of male nursing students in Taiwan. Confirmatory factor analysis was used to select items and to confirm the scale’s reliability and validity. In addition, we used latent multiple regression analyses to determine the background variables that influence EI and its four factors. We found that, although there were no gender differences in the EI total scale factor, male nursing students had significantly higher self-emotional expression, but lower self-emotional management, than female students. Furthermore, age was significantly negatively associated with self-emotional management for both genders. In addition, having religious beliefs was significantly associated with lower recognition of others’ emotions, emotional self-awareness, self-emotional expression, and the EI total scale factor; it was particularly significantly associated with lower emotional self-awareness in male students and lower recognition of others’ emotions in females. Finally, father’s education level, rather than mother’s, in general, had a positive association with the EI total scale factor and all its four subfactors. For male students, father’s and mother’s education levels were not associated with any EI factors, while for females, father’s education level had a significantly positive association with the EI total scale factor and its four subfactors; however, mother’s education level had a significantly negative association with recognizing the emotions of others and self-emotional expression. 

## Figures and Tables

**Table 1:** Demographic characteristics of the participants (*n* = 786).

Background Variables	*n*	%
Gender		
Male	384	48.9
Female	402	51.1
Religious beliefs		
No	290	36.9
Yes	496	63.1
Father’s education level		
Elementary school	58	7.4
Junior high school	148	18.8
Senior high school/Vocational school	296	37.7
Junior college	137	17.4
University	90	11.5
Graduate school and above	57	7.3
Mother’s education level		
Elementary school	53	6.7
Junior high school	135	17.2
Senior high school/Vocational school	294	37.4
Junior college	147	18.7
University	104	13.2
Graduate school and above	53	6.7

**Table 2:** Factor loadings and reliability coefficients of the EI scale (*n* = 384; male nursing students).

Items	Factor Loading	Cronbach’s Alpha
1. I can recognize others’ emotions from their behaviors	0.80	
2. I can recognize others’ emotions from their body posture	0.75	
3. I can recognize others’ emotions from their tone	0.75	
4. For me, it is meaningful to know others’ emotions	0.71	0.84
5. In tense moments, I am usually aware of my emotions	0.69	
6. When in an unstable emotional state, I am immediately aware of it	0.73	
7. When in a negative emotional state, I know the reason for it	0.76	
8. On most occasions, I am aware of whether or not my emotional responses are appropriate	0.68	0.81
9. When facing conflicts, I can use proper words to express emotional states	0.71	
10. I can adequately explore my emotional states with others	0.73	
11. I can use proper gestures to express my thoughts	0.71	
12. When facing conflicts, I am willing to help relieve others’ emotions	0.69	0.80
13. On tense occasions, I think of ways to relieve emotions	0.72	
14. When feeling anxious, I think of ways to calm down	0.75	
15. I try to stay serene in all circumstances	0.77	
16. When something contrary to my wishes occurs, I try to put it down first	0.64	0.81

Note: “recognizing the emotion of others” includes items 1−4, “emotional self-awareness” includes items 5−8, “self-emotional expression” includes items 9−12, and “self-emotional management” includes items 13−16. The Cronbach’s alpha coefficient for the total scale was 0.93. The means (standard deviations) were 14.27 (3.51), 14.53 (3.48), 14.29 (3.52), 13.54 (3.59) for each subscale, respectively, and 56.63 (12.13) for the total scale.

**Table 3:** Latent multiple regression of the EI total scale factor and its four subfactors on gender, age, religious beliefs, and father’s and mother’s education levels.

	Scale Dimensions				
Variables	Recognizing the Emotions of Others	Emotional Self-Awareness	Self-Emotional Expression	Self-Emotional Management	EI Total Scale Factor
Constant	1.377 (0.830)	1.349 (0.820)	1.275 (0.676)	3.052 *** (0.724)	1.678 * (0.692)
Gender	0.089 (0.092)	0.144 (0.091)	0.189 * (0.075)	−0.227 ** (0.080)	−0.047 (0.072)
Age	−0.039 (0.039)	−0.043 (0.039)	−0.050 (0.032)	−0.124 *** (0.034)	−0.060 (0.031)
Religious beliefs	−0.196 * (0.094)	−0.230 * (0.093)	−0.158 * (0.077)	−0.147 (0.082)	−0.156 * (0.074)
Father’s education level	0.204 *** (0.046)	0.142 ** (0.045)	0.152 *** (0.037)	0.107 ** (0.040)	0.135 *** (0.036)
Mother’s education level	−0.082 (0.046)	0.028 (0.046)	−0.002 (0.038)	0.011 (0.040)	−0.009 (0.036)

Note: * *p* < 0.05, ** *p* < 0.01, *** *p* < 0.001. The regression coefficients are shown in cells with standard errors in parentheses. The female group is the reference group for gender differences, and no religious belief is the reference group for religious beliefs. *n* = 786 nursing students.

**Table 4:** Latent multiple regression of the EI total scale factor and its four factors on background variables, separately for both genders.

	Dimensions				
Variables	Recognizing the Emotions of Others	Emotional Self-Awareness	Self-Emotional Expression	Self-Emotional Management	EI Total Scale Factor
**Male students**					
Constant	2.893 (2.051)	2.607 (1.661)	2.009 (1.544)	3.280 * (1.566)	2.354 (1.437)
Age	−0.106 (0.096)	−0.103 (0.078)	−0.074 (0.073)	−0.153 * (0.074)	−0.098 (0.067)
Religious beliefs	−0.182 (0.178)	−0.329 * (0.144)	−0.251 (0.134)	−0.135 (0.136)	−0.183 (0.123)
Father’s education level	0.163 (0.086)	0.111 (0.069)	0.051 (0.065)	0.089 (0.066)	0.092 (0.059)
Mother’s education level	−0.011 (0.088)	0.116 (0.071)	0.126 (0.066)	0.094 (0.067)	0.074 (0.061)
**Female students**					
Constant	1.019 (0.689)	0.982 (0.795)	1.237 (0.686)	2.870 *** (0.701)	1.470 *(0.670)
Age	−0.021 (0.033)	−0.024 (0.038)	−0.051 (0.032)	−0.111 *** (0.033)	−0.051 (0.032)
Religious beliefs	−0.194 * (0.092)	−0.098 (0.106)	−0.051 (0.091)	−0.169 (0.093)	−0.125 (0.088)
Father’s education level	0.212 *** (0.045)	0.163 ** (0.051)	0.238 *** (0.044)	0.132 ** (0.045)	0.169 *** (0.043)
Mother’s education level	−0.127 ** (0.044)	−0.053 (0.051)	−0.105 * (0.044)	−0.069 (0.045)	−0.077 (0.043)

Note: * *p* < 0.05, ** *p*< 0.01, *** *p*< 0.001. The regression coefficients are in cells with standard errors in parentheses. “No religious belief” is the reference group for “religious beliefs”.

## Data Availability

The data presented in this study are available on request due to privacy restrictions.
